# Monitoring Irrigation in Small Orchards with Cosmic-Ray Neutron Sensors

**DOI:** 10.3390/s23052378

**Published:** 2023-02-21

**Authors:** Cosimo Brogi, Vassilios Pisinaras, Markus Köhli, Olga Dombrowski, Harrie-Jan Hendricks Franssen, Konstantinos Babakos, Anna Chatzi, Andreas Panagopoulos, Heye Reemt Bogena

**Affiliations:** 1 Agrosphere Institute (IBG-3), Forschungszentrum Jülich GmbH, 52425 Jülich, Germany; 2 Soil & Water Resources Institute, Hellenic Agricultural Organization, Gorgopotamou, Sindos, 57400 Thessaloniki, Greece; 3 Physikalisches Institut, Heidelberg University, 69120 Heidelberg, Germany

**Keywords:** cosmic-ray neutron sensor CRNS, irrigation management, soil moisture monitoring, apple orchard

## Abstract

Due to their unique characteristics, cosmic-ray neutron sensors (CRNSs) have potential in monitoring and informing irrigation management, and thus optimising the use of water resources in agriculture. However, practical methods to monitor small, irrigated fields with CRNSs are currently not available and the challenges of targeting areas smaller than the CRNS sensing volume are mostly unaddressed. In this study, CRNSs are used to continuously monitor soil moisture (SM) dynamics in two irrigated apple orchards (Agia, Greece) of ~1.2 ha. The CRNS-derived SM was compared to a reference SM obtained by weighting a dense sensor network. In the 2021 irrigation period, CRNSs could only capture the timing of irrigation events, and an ad hoc calibration resulted in improvements only in the hours before irrigation (RMSE between 0.020 and 0.035). In 2022, a correction based on neutron transport simulations, and on SM measurements from a non-irrigated location, was tested. In the nearby irrigated field, the proposed correction improved the CRNS-derived SM (from 0.052 to 0.031 RMSE) and, most importantly, allowed for monitoring the magnitude of SM dynamics that are due to irrigation. The results are a step forward in using CRNSs as a decision support system in irrigation management.

## 1. Introduction

Agricultural practices are responsible for ~80% of the fresh water that is consumed globally [1]. A considerable portion of this consumption represents water collected from rivers, lakes, and aquifers that are then used for irrigation [2]. In the south–eastern Mediterranean region especially, irrigation demand is higher than the global average due to scarce summer precipitation, overexploitation of water resources, and climate change [3,4,5,6,7]. A key to relieve the water demand in this area, and in arid and semi-arid regions in general, is improving water use efficiency by informing irrigation practices. This can be achieved, for example, by early detection of canopy stress [8,9] or by monitoring spatio-temporal soil moisture (SM) variations [10,11,12]. The latter can be achieved using soil moisture sensors. However, commonly used sensors and sources of SM information are either point-scale and invasive (e.g., time domain reflectometry, frequency domain reflectometry, and direct soil sampling) or remote sensing products (e.g., passive, and active microwave sensors) [13,14,15,16,17]. Both have drawbacks: the former only provide local information [18,19,20], whereas the latter are affected by land surface characteristics, have penetration depth of only a few cm, and typically have limited temporal and/or spatial resolution [21,22,23].

Recently, promising results in bridging the gap between point-scale and remote sensing products have been shown by cosmic-ray neutron sensors (CRNSs) [24]. This technique measures the environmental neutron density produced by cosmic radiation. As such, neutron density is inversely related to below- and aboveground hydrogen pools, and can be used to estimate SM [25,26,27]. CRNSs generally detect neutrons that are in the thermal (below 0.5 eV) or epithermal (0.5 eV to 0.5 MeV) energy regimes [28], but the sensitivity towards the more SM-sensitive epithermal neutrons can be enhanced by using a high-density polyethylene (HDPE) moderator, or by adding a thermal shield [26,29,30]. The unique advantage of CRNSs lays in the large volume investigated [31,32]. A CRNS can generally measure over a radius of 120 to 240 m and up to 15 to 85 cm in depth [33]. Both the measured radius and depth can vary depending on environmental variables, such as vegetation, humidity, and SM [33,34,35]. In addition, CRNSs typically require low maintenance, are non-invasive, provide continuous measurements [36], and are not affected by soil temperature [37] or soil chemistry [27]. Especially in agricultural applications, CRNSs present less logistic challenges than, for example, networks of sensors that are installed in the soil. As a CRNS can be placed out of the way of management practices, it is generally not necessary to remove such a sensor during harvesting, planting, and other managements [38].

As CRNSs are still relatively new instruments, recent studies and developments have focused on the various processes that affect this measurement technique. Generally, SM conditions have the largest effect on measured neutron intensities. Additionally, more accurate measurements are obtained in dry soils compared to wet soils, as the former show higher environmental neutron density that results in higher neutron counts measured by the CRNS [26,39]. Furthermore, snow cover has a strong effect on CRNS measurements [40], which allows for monitoring snowpack dynamics and informing snow hydrological models [41,42,43]. To a lesser degree, CRNSs are also affected by vegetation [44,45], atmospheric water vapor [46], intercepted water in the canopy, lattice water [39], and horizontal heterogeneities [47,48,49]. The range of CRNS applications has steadily increased since its introduction. For example, due to the large measured area, CRNSs have been used to validate and support satellite-based SM products [50,51], hydrological models [52], land surface models [53,54], and the study of vegetation dynamics [44,55]. Finally, it is expected that the use of CRNSs will increase in the future thanks to the developments in coverage, data availability in near-real time, decreasing costs, and the possibility to perform rover-based measurements [56,57,58].

In the context of agricultural management, CRNSs have the potential to monitor and inform irrigation practices [59]. For example, CRNSs can monitor SM deficit in the root zone [60], and can be combined with electrical conductivity surveys to improve water use efficiency in large pivot-irrigated fields [37]. In flooding irrigation, Ref. [61] used CRNS-derived SM to monitor four irrigation events, and successfully analysed the field water use efficiency of an experimental field in the Shaanxi province (China). In a similar agricultural context in the Zhangye oasis (China), Ref. [62] showed that CRNS-derived SM is comparable to SM products obtained by sensor networks, but observed a general SM overestimation due to ponding water. Moreover, Ref. [63] showed that CRNSs react to sprinkler irrigation timing, but could not clearly quantify the response produced by single irrigation events. These studies, however, generally focused on large, irrigated areas and on irrigation techniques that result in widespread SM variation. In the context of drip irrigation, a synthetic study by [64] suggested that data assimilation of CRNS neutron intensities could improve modelled SM estimates and irrigation scheduling, in an orchard in Spain. However, in the same study area, Ref. [65] subsequently found that it was not possible to accurately monitor drip irrigation with a standard CRNS, due to the small dimension of the wetted area, small variations in SM, and insufficient neutron count and SM sensitivity of the specific instrument that was employed. These studies show that it is not yet possible to clearly assess the usability of CRNSs as a precise irrigation tool that informs farmers on the actual SM of their field, and on the need for irrigation in terms of timing and quantity. Moreover, the recently introduced concept of sub-footprint heterogeneity [42,49] was rarely considered in these applications, although it is key for small, irrigated fields, surrounded by either non-irrigated land or different irrigation managements [35]. Further developments are thus needed to establish practical implementations of CRNSs that are an added value for stakeholders [59], especially in irrigation management.

Within this context, this study investigates the possibility of using CRNSs to continuously monitor the timing and magnitude of SM dynamics in two irrigated apple orchards in Agia (Greece). A dense network of sensors that recorded meteorological variables, irrigation timing and quantity, and SM at multiple depths and locations, was installed alongside two CRNSs. To evaluate the performance of the CRNSs, the CRNS-derived SM was compared to reference SM obtained from weighting the SM measurements of the sensor network. For the 2021 irrigation period, a standard CRNS calibration method was compared with an ad hoc calibration during the irrigation period, as well as one, six, and twelve hours before irrigation. Recently, it has been shown that filtering of CRNS data can affect the quality of the soil moisture product [66]. Therefore, different options were investigated for the ad hoc calibration case. For the 2022 irrigation period, an additional SM sensor was located outside one of the investigated fields, and neutron transport simulations were performed to obtain information on the detected neutron origins. This allowed for the development and testing of a correction procedure based on neutron transport simulations, to obtain a CRNS-derived SM that better represents the SM in small, irrigated fields.

## 2. Materials and Methods

### 2.1. Study Area, Soil Sampling, and Installed Instrumentation

**Figure 1:**
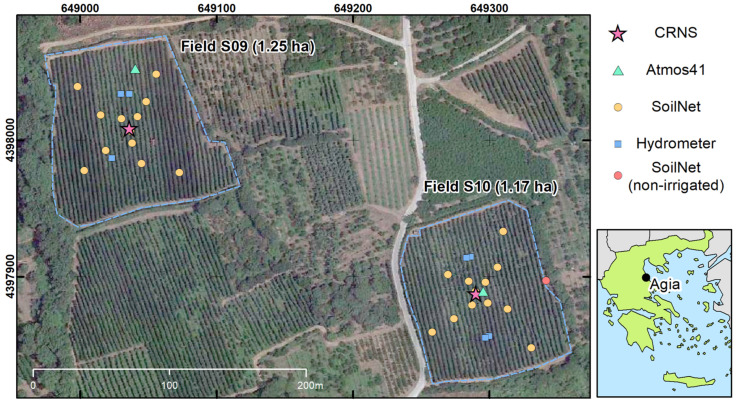
Satellite image [75] of the study area, instrumentation, and land use. Reference system is UTM 1984 Zone 34 North.

The study area was located near Agia (Greece) and was part of the Pinios Hydrologic Observatory (PHO) [67], which belongs to the Pinios River basin and is among the most productive agricultural areas in Greece. The PHO is a member-site of the Hellenic and International long-term ecosystem research (LTER) network. It is characterized by Mediterranean climate, with annual average temperatures of 15 °C and yearly precipitation amounts between 500 and 1200 mm. Irrigation is commonly employed in the PHO due to the generally scarce summer precipitation, and irrigation water demand is generally met by groundwater abstraction [68]. Within the PHO, two apple orchards were selected for this study, namely field S09 and field S10 (Figure 1). These fields are part of the pilot fields established in the context of the ATLAS project (https://www.atlas-h2020.eu/, 29 December 2022) and are used for the development of data-driven irrigation services. They are part of the Oikonomou farm, which holds ~7 ha of apple orchards in the area. The investigated fields had a mild south-oriented slope (<5%), and areas of 1.25 ha (field S09) and 1.17 ha (field S10). The area that surrounded the investigated fields was characterized by a gentle and constant slope (generally < 7%), with a north–south orientation. Steeper slopes were only found 400 m northwest of field S09 or beyond. Both fields were equipped with micro sprinkler irrigation systems and were irrigated weekly, generally towards the end of each week.

At each field, 15 sampling locations were selected and visited in autumn 2020. At each location, soil samples at 0 to 30 cm, 30 to 60 cm, and 60 to 100 cm depth were collected using an Edelman auger. For each sample, the fractions of gravel, sand, silt and clay, and the soil organic matter content were estimated. The former was estimated using the Bouyoucos method [69,70], while the latter was obtained with the Walkley–Black method [71]. The bulk density was obtained from gravel content using the method of Brakensiek and Ralws [72] (see Appendix A for additional details), and the lattice water was obtained from clay content using the relationship of Dong et al. [57]. In each field, the textural and bulk density information at each sampling depth were averaged and used to estimate soil hydraulic parameters using the pedotransfer functions (PTFs) of Rawls and Brakensiek [73] (see Appendix A). These soil hydraulic parameters were used to reconstruct water retention curves in each field by using the Mualem–van Genuchten model [74].

The two investigated fields were equipped with an extensive network of sensors to monitor irrigation practices, meteorological variables, and SM dynamics. Most of the instruments were installed between 5 and 9 September 2020. At each field, an Atmos 41 all-in-one weather station (Meter Group AG, Munich, Germany) was installed as close as possible to the field centre. In the case of field S09, the Atmos 41 was installed ~40 m north of the field centre (Figure 1), as this was the closest position where a stable pole could be installed. The Atmos 41 stations provided measurements of precipitation, air temperature, relative humidity, and atmospheric pressure. A backup rain gauge (Vaisala WXT520), installed ~3800 m from the target fields (39°43′53.61″ N, 22°43′29.50″ E), was used to confirm the reliability of the precipitation measurements provided by the Atmos 41 sensors. Moreover, seven water meters (TECNIDRO TW-N s. r. l., Genova, Italy) were installed on 24 May 2021, at the start of the irrigation period: three in field S09 and four in field S10. In field S09, the water meters were installed in the three most representative irrigated sections (out of five sections in total). In S10, all four irrigated sections were monitored. In each field, the measured volume of irrigation water was converted in mm using the area of the monitored irrigated sections. To monitor SM dynamics, each field was equipped with a SoilNet wireless sensor network consisting of 12 nodes [18]. Each node was equipped with six SMT100 (Truebner GmbH, Neustadt, Germany) installed at 5, 20, and 50 cm depth, in two separate profiles. The two profiles were positioned along a tree row and had a separation of 1.5 m. This setup was selected to mitigate the effects of the heterogeneous irrigation patterns created by the mini sprinklers in the target fields. Prior to installation, each sensor was calibrated according to [76]. On 16 September 2021, an additional SoilNet node was installed in a non-irrigated “dry spot” outside field S10, to monitor SM dynamics outside the irrigated area.

Finally, two CRNSs (Styx Neutronica GmbH, Mannheim, Germany) were installed in the centre of fields S09 and S10. These CRNSs use a 25 mm high-density polyethylene (HDPE) moderator and can host up to five counter tubes that are based on solid boron carbide (B_4_C) coating [30,77]. In this study, each CRNS was equipped with two counter tubes. However, the CRNS in field S09 only had one active counter tube until 6 April 2022. Both instruments were equipped with a gadolinium oxide (Gd_2_O_3_) shield, that could absorb more than 90% of thermal neutrons [29,30,77].

All instruments that were installed in the target fields provided near-real time measurements with a temporal resolution of 15 min, that were later aggregated to hourly time steps (daily time steps in the case of precipitation or irrigation). The water meters used LoRaWAN transmission loggers (Delta-OHM LR35, Italy), whereas the other instruments used NB-IoT technology.

#### 2.1.1. In-Situ Soil Sampling for CRNS Calibration

In-situ soil samples were collected to calibrate the two CRNSs according to the sampling scheme proposed by Schrön et al. As suggested by [34], where the stronger influence of neutrons that originate nearby the CRNS [27] was taken into account. Samples for calibration were collected on August 30th, 2021. As a generally wet condition was expected in the irrigated target fields, 18 sampling locations, divided in three radial distances of 2, 25, and 85 m, were selected [34]. In both fields, the 85 m distance was outside the irrigated area. For the 85 m distance of field S09, it was only possible to visit five locations and, thus, only 17 soil samples were collected. A HUMAX soil corer (Martin Bruch AG, Rothenburg, Switzerland) was used to retrieve soil cores up to 30 cm depth. Each core was divided into segments of 5 cm and the water content was determined by oven drying at 105 °C for 24 h. Bulk density and lattice water were not directly estimated using the HUMAX soil core, but were obtained from the closest available Edelman sampling location at 0 to 30 cm depth.

#### 2.1.2. Reference SM from SoilNet Data

A recent method for vertical and horizontal weighting [34] was used to obtain a reference SM value, with an hourly resolution from the SoilNet network of each of the two target fields. This method is based on the fact that the CRNS footprint changes due to variations in SM and in other environmental variables [33]. First, vertically weighted SM values for each SoilNet location were derived and then averaged using location-specific horizontal weight that depends on the distance from the CRNS. A detailed description of this method and its applications can be found in [34]. In the case of field S09, weighting of the SoilNet network was used to obtain a second calibration on 10 April 2022, during relatively homogeneous SM conditions in the field and surroundings. Such additional calibration was necessary as, after maintenance, the CRNS of field S09 had two active counter tubes instead of one. Furthermore, weighting of the SoilNet network on 2 September 2021 was used to obtain an ad hoc calibration as an alternative to the standard calibration approach for both fields.

### 2.2. Monte Carlo Simulations of Neutron Transport

To obtain information on the origin of neutrons detected by the CRNSs, simulations of neutron transport and their interactions with matter were performed with the Monte-Carlo-based URANOS [78] model (http://www.ufz.de/uranos, 29 December 2022). In URANOS, a large number of simulated neutrons, and thus relatively accurate simulation results, can be achieved thanks to the high computational efficiency of effective models that represent processes within atmospheric cascades. This ultimately renders URANOS suited for environmental research [33]. Neutrons are emitted from randomly distributed points in a source layer defined by the user, where energies are sampled from a validated spectrum above the ground [79]. The propagation of a neutron and its physical interactions (e.g., elastic collisions, inelastic collisions, absorption, and emission processes such as evaporation) are then tracked with a standard calculation routine, featuring a ray-casting algorithm.

#### 2.2.1. Neutron Origins from Existing URANOS Simulations

A dataset of 500 URANOS simulation results from [35] was used to retrieve a synthetic subdivision between detected neutrons N that originate (a) inside the target field ($N_{in}$), (b) outside the target field ($N_{out})$, and (c) that had no contact with soil ($N_{non-albedo}$). This was performed by using the energy-dependent response function of CRNSs, with a 25 mm HDPE moderator and a gadolinium shield [29,30]. The available simulations include 0.5, 1, 2, 4, and 8 ha fields, with SM combinations that range from 0.05 to 0.50 cm^3^ cm^−3^ inside and outside the target field. The results of these simulations were interpolated using cubic spline functions. Subsequently, $N_{in}$, $N_{out}$, and $N_{non-albedo}$ were obtained for two synthetic square fields, with areas equal to that of fields S09 and S10 (i.e., 1.25 and 1.17 ha, respectively). The SM values for the area surrounding these fields were selected from the observed SM variations of the SoilNet in the dry spot (Figure 1). The SM inside each field was set equally to the SM at field capacity (−100 cm) and at wilting point (−15,000 cm), as indicated by the water retention curve of each field at 0 to 30 cm depth.

#### 2.2.2. Neutron Origins from URANOS Simulations of the Study Area

New URANOS simulations, tailored to fields S09, S10 and to their surroundings, were performed to obtain $N_{in}$, $N_{out}$, and $N_{non-albedo}$ from a more realistic representation of the spatial and vertical distribution of SM and vegetation of the study area. These were later compared to the use of $N_{in}$, $N_{out}$, and $N_{non-albedo}$ obtained from the simulations of [35]. In these new simulations, 10^8^ neutrons were simulated, while the domain extended over a 144 ha area (1200 × 1200 m), and had nine atmospheric and three soil layers. The land surface was assumed to be flat, as the study area only shows gentile and constant north–south-oriented slopes in the vicinities of the CRNSs. The atmospheric layers, starting from 1000 m above the soil surface, extended for 920.0, 30.0, 44.0, 2.0, 1.5, 0.5, 1.0, 0.9, and 0.1 m. The second and the sixth atmospheric layers were set as source and detector layers, respectively. The soil layer thicknesses were 0.3, 0.3, and 1.0 m (maximum 1.6 m depth). The simulation domain was divided in eight land-cover classes, that were digitized from a satellite image [75] using the ArcGIS software (ArcGIS Desktop: Release 10.7.1, Redlands, CA: Environmental Systems Research Institute). The digitized map was transformed into a 1 m resolution raster using the same software package. Atmospheric layers four to nine had a biomass that depended on four of the eight land-use classes. Such a biomass was set equal to the materials code *tree gas* (3.0 g cm^−3^, see software manual) of the URANOS software in the case of an irrigated orchard and of natural trees. The *plant gas* (5.0 g cm^−3^) material code was used for bush and grass. Asphalt roads and buildings were simulated using their respective material codes in the URANOS software. Although the natural trees that are present in the domain were of very different species and geometries, they were assumed to have a constant height of 6 m. The height of the other land covers was set to 4 m for orchard trees, 0.6 m for bushes, 0.1 m for grass, 0.1 m for asphalt roads, and 4 m for buildings. This representation of the vegetation cover in the neutron transport simulations is rather simplified. However, vegetation cover can be considered less important in this case, as the CRNSs used in this study employed a 25 mm HDPE moderator with gadolinium shielding, and thus less than 10% of the detected neutrons fall into the thermal, more biomass-dependent energy range [47,55,77,80].

The maximum and minimum SM values for the three soil layers in the non-irrigated surroundings of the investigated fields were selected from the observed SM variations of the SoilNet in the non-irrigated spot (Figure 1). In the irrigated fields, the maximum and minimum SM were set equally to the SM at field capacity (−100 cm) and at wilting point (−15,000 cm), as indicated by the water retention curves of each field at 0 to 30, 30 to 60, and 60 to 100 cm depths. In the case of the irrigated fields surrounding the target fields, no information on SM or soil physical properties was available. Thus, they were assumed to have a SM equal to the average between fields S09 and S10. This is a rather simplified approach, but it should be noted that such irrigated fields are located at distances between 80 and 600 m from the CRNS, and only cover a portion of the simulated domains. Thus, a small variation in the SM of such surrounding fields is not expected to have a decisive influence on the CRNS detection.

### 2.3. CRNS-Derived SM and Novel Correction for Small Irrigated Fields

#### 2.3.1. Neutron Count Correction and Conversion to SM Content

As the measured incoming neutron intensities can be influenced by various factors, measured neutron counts were first aggregated to hourly time steps and then processed according to [81]. First, raw neutron counts ($N_{raw}$) below 50 and above 2000 cph (counts per hour) were removed. The latter value was used instead of the value reported in [81] (10,000), as it was unlikely that the detectors used in this study would achieve a cph > 2000 in the investigated environment. Then, $N_{raw}$ higher than a 24-h moving average, plus twice the standard deviation of the 24 h rolling average, were removed. The same procedure was applied to $N_{raw}$ lower than a 24 h moving average, minus twice the standard deviation of the 24 h rolling average.

The resulting measured neutron counts were used to obtain the corrected neutron count N by applying a set of correction factors:

(1) $$N=N_{raw}*\;C_{p}*C_{h}*C_{i}*C_{v},$$

where $C_{p}$ [82], $C_{h}$ [46], $C_{i}$ [83], and $C_{v}$ [45] are the atmospheric pressure, air humidity, incoming neutron, and biomass correction factors, respectively [82]. These correction factors are obtained from:

(2) $$C_{p}=e^{ß\left(P-P_{0}\right)},$$

(3) $$C_{h}=1+\alpha h,$$

(4) $$C_{i}=[1+\gamma {\left(I/I_{ref}-1\right)]}^{-1},$$

(5) $$C_{v}={\left[1-bB\right]}^{-1},$$

where P and $P_{0}$ are the actual and reference atmospheric pressure (hPa), respectively, ß is a barometric coefficient assumed to be equal to 0.0076 hPa^−1^, h is the absolute humidity (g m^−3^), α equals to 0.0054 m^3^ g^−1^, I is the incoming count rate of the Jungfraujoch neutron monitor (Switzerland), $I_{ref}$ is the incoming count rate at an arbitrary time, γ is a scaling factor that adjusts geomagnetic effects [84], B is the dry biomass (kg m^−2^), and b equals to 0.009248 m^2^ Kg^−1^. The biomass for an apple orchard was obtained from literature values [85]. Although biomass changes can be observed during a growing season, we assumed a constant biomass, as changes in the target fields were not abrupt (e.g., several weeks long harvest) or did not result in the removal of large biomass fractions (e.g., trimmed branches and fallen leaves are left in the field).

Finally, a centred running 24 h average of the measured neutron count rate was calculated to reduce noise and measurement uncertainty [27]. Additionally, centred running averages with 12 h and 6 h window sizes were produced. For these three window sizes, right-aligned rolling averages were calculated, as well. In both field S09 and S10, a comparison of the results obtained with different running averages was made at one, six, and twelve hours before irrigation events in 2021. The neutron count rate N was converted into SM values $\theta \left(N\right)$, by applying the relationship introduced by [26]:

(6) θN=QBDα0NN0−α1−1−α2−θoff,

where α0, α1, α2 are parameters that are set to 0.0808, 0.372, and 0.115, respectively, $N_{0}$ is the count rate over dry soil, $Q_{BD}\;$is the bulk density, and $\theta_{off}$ is the gravimetric soil moisture equivalent converted from lattice water and organic matter [48]. For the 2021 irrigation period, a standard CRNS calibration method was compared with an ad hoc calibration during the irrigation period, as well as one, six, and twelve hours before irrigation. For the second irrigation period, an additional SM sensor was located outside one of the investigated fields. This allowed to develop and test a correction procedure based on neutron transport simulations, to obtain a CRNS-derived SM that better represents the SM in small, irrigated fields.

#### 2.3.2. Novel CRNS Correction for Small, Irrigated Fields

**Figure 2:**
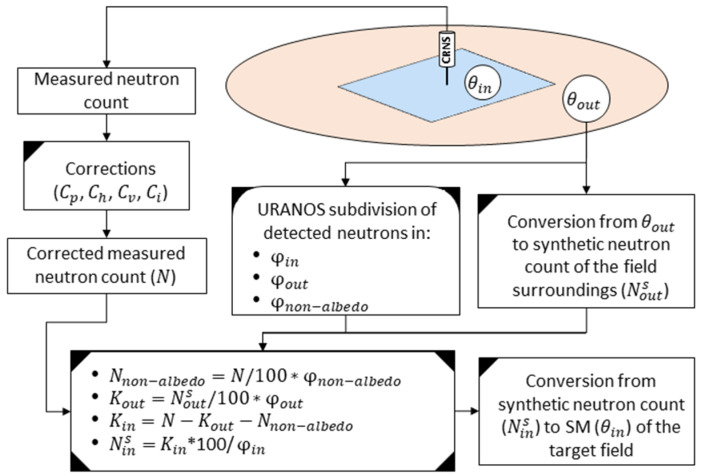
Flowchart of the proposed CRNS correction method to obtain $\theta_{in}$.

In this study, a correction of the CRNS-derived SM for small, irrigated fields is developed by combining novel and existing concepts, such as CRNS spatial sensitivity, sub-footprint heterogeneity, and the spatial origin of detected neutrons [34,35,47,49]. A flowchart of the general methodology, that was used to correct CRNS-derived SM by using SM measured outside the irrigated field, is depicted in Figure 2. Additionally, a spreadsheet named “CRNS_Correction_DIY” (Supplementary Materials), where the methods and equations are shown with some examples, is made available as supplementary material.

For a CRNS located in the centre of a small, irrigated field, the corrected count rate N can be approximately subdivided in:

(7) $$N=N_{in}+N_{out}+N_{non-albedo},$$

where $N_{in}$ is the detected neutrons that originate (have first soil contact) in the target irrigated field, $N_{out}$ is the detected neutrons that originate in the surroundings, and $N_{non-albedo}$ is the detected non-albedo neutrons (that have no soil contact). This subdivision can be obtained using neutron transport simulations, and depends on the area of the irrigated field, as well as on the SM value in the irrigated field ($\theta_{in}$) and in the surroundings ($\theta_{out}$) [35]. A neutron transport simulation will thus result in three percentages φ:

(8) $$100\%=\varphi_{in}+\varphi_{out}+\varphi_{non-albedo},$$

where $\varphi_{in}$ is the percentage of the detected neutrons that originate in the target field, $\varphi_{out}$ is the percentage of the detected neutrons that originate in the surroundings, and $\varphi_{non-albedo}$ is the percentage of the detected neutrons that have no soil contact.

In the example of Figure 2, a CRNS is placed in the middle of a small irrigated field and the SM values within the irrigated field ($\theta_{in}$) and in the surroundings ($\theta_{out}$) are assumed to be homogeneous in their respective areas. The subdivision of N in $\varphi_{in}$, $\varphi_{out}$, and $\varphi_{non-albedo}$, for this scenario, is obtained with neutron transport simulations. Then, using Equation (6) inverted, a synthetic neutron count can be estimated from $\theta_{out}$ (i.e., $N_{out}^{s}$). Ideally, this synthetic $N_{out}^{s}$ is the neutron count that the CRNS would measure if placed in an infinite area with a SM equal to $\theta_{out}$.

Once $\theta_{out}$ (and thus $N_{out}^{s}$), $\varphi_{in}$, $\varphi_{out}$, and $\varphi_{non-albedo}$ are known, the proposed correction method involves the following steps:

- First, $\varphi_{non-albedo}$ is used to calculate the portion of N that is composed of non-albedo neutrons using: $N_{non-albedo}=N/100*\varphi_{non-albedo}$;
- Then, a $K_{out}$ coefficient, that is proportional to the $N_{out}^{s}$ that would be measured if the CRNS was solely surrounded by a SM equal to $\theta_{out}$, is obtained using: $K_{out}=N_{out}^{s}/100*\varphi_{out}$;
- In the following step, a $K_{in}$ coefficient, that is proportional to the $N_{in}^{s}$ that would be measured if the CRNS was solely surrounded by a SM equal to $\theta_{in}$, is obtained using: $K_{in}=N-K_{out}-N_{non-albedo}$;
- Finally, the synthetic neutron count $N_{in}^{s}$ is obtained using $N_{in}^{s}=K_{in}*100/\varphi_{in}$. This is then used in Equation (6) to estimate the SM in the irrigated field ($\theta_{in}$).

Ideally, the described method is better used when both $\theta_{in}$ and $\theta_{out}$ are known for a target field. However, in irrigation monitoring, these SM values are generally unknown. To assist the CRNS placed in an irrigated field, a single SM sensor (e.g., a SoilNet node) can be installed outside the field to measure $\theta_{out}$ (and thus estimate $N_{out}^{s}$). In the case of $\theta_{in}$, such a SM value was assumed to be unknown in this study to allow for an evaluation of the performance of the proposed procedure. As $\varphi_{in}$, $\varphi_{out}$, and $\varphi_{non-albedo}$ are influenced by both $\theta_{in}$ and $\theta_{out}$, maximum and minimum values for $\theta_{in}$ and $\theta_{out}$ must be assumed. In the case of $\theta_{out}$, maximum and minimum values (i.e., $\theta_{out\left(max\right)}$ and $\theta_{out\left(min\right)}$) can be observed with the above-mentioned SM sensor during a test period. To obtain $\theta_{in\left(\max\right)}$ and $\theta_{out\left(max\right)}$ instead, a water retention curve, obtained from laboratory analysis of soil samples (as in this study) or existing datasets of soil properties, could be used. Once these SM values are selected, four different neutron transport simulations, with SM combinations as described in Table 1, are produced.

The neutron transport simulations, performed using SM values from Table 1, provide four different sets of $\varphi_{in}$, $\varphi_{out}$, and $\varphi_{non-albedo}$. In a first iteration, a dry condition of the irrigated field is assumed. In this case, $\varphi_{in}$, $\varphi_{out}$, and $\varphi_{non-albedo}$ are obtained by linearly interpolating the results of the dry–dry and dry–wet neutron transport simulations (Table 1), and by subsequently calculating the values of $\varphi_{in}$, $\varphi_{out}$, and $\varphi_{non-albedo}$ that correspond to $\theta_{out}$ measured outside the irrigated field. The results are used to estimate a minimum $\theta_{in\left(min\right)}$. In a second iteration, a wet condition of the irrigated field is assumed, and results from wet–dry and wet–wet simulations (Table 1) are used to obtain $\varphi_{in}$, $\varphi_{out}$, and $\varphi_{non-albedo}$ depending on $\theta_{out}$. These are used to estimate a maximum $\theta_{in\left(max\right)}$. Finally, $\theta_{in\left(min\right)}$ and $\theta_{in\left(max\right)}$ will represent a range of SM values for the irrigated field, and can be averaged to obtain a CRNS-derived SM that is corrected for the influence of SM outside the target irrigated field.

## 3. Results and Discussion

In the following, the variables measured in the two investigated fields, S09 and S10, are discussed, and the CRNS-derived SM is compared with the reference SM obtained from the SoilNet networks. In this comparison, it should be noted that reference the SM was obtained from SoilNet nodes that are solely located within the irrigated field. Thus, the reference SM was not influenced by the field surroundings. Given that, for the CRNSs used in this study, the sensing volume was larger than the relatively small target fields (1.17 and 1.25 ha), a large part of the detected neutrons that have soil contact can be expected to originate in the surroundings. This has an influence on the measured neutron count rates, and a direct comparison between CRNS-derived SM and the selected reference could be considered unbalanced, especially when SM inside and outside the irrigated fields differ considerably. However, the scope of this study is to test CRNS for the retrieval of SM information within the irrigated field, which justifies the use of the selected methodologies and comparisons, even if the CRNS is put in a generally unfavourable testing environment.

### 3.1. Measurements in the Target Fields and CRNS-Derived SM

**Figure 3:**
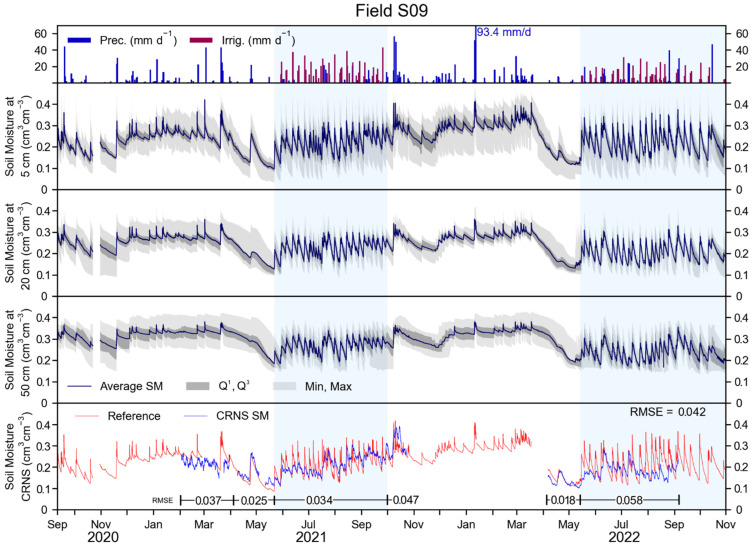
In the upper panel: precipitation for field S10, and irrigation for field S09 in mm d^−1^. In the three central panels: SM recorded from the SoilNet network of field S09 at 5, 20, and 50 cm depths. Dark grey areas indicate the first and third quartile, whereas the light grey area indicates maximum and minimum values for the SM of these panels. In the bottom panel: reference SM from weighting the SoilNet network and CRNS-derived SM for field S09. Regressions of these measurements are provided in Appendix C.

The daily precipitation for field S10 and irrigation for field S09 are shown in Figure 3 (top panel). Here, the precipitation measurements of S10 are shown, since they are 50.7% higher than those provided by the Atmos 41 sensor in field S09 (not shown). This large discrepancy was attributed to erroneous measurements from the Atmos 41 installed in S09. In fact, lower precipitation measurements can occur between neighbouring sensors [86] and can be exacerbated, for example, by the clogging of the rain gauge. Moreover, the measurements in field S10 better match those recorded at the backup rain gauge (not shown), and were thus used in place of those of field S09. The 2021 irrigation period started on 15 May and ended on 26 September, whereas the 2022 period started on 14 May and ended on 27 October. In general, irrigation was applied during one to three consecutive days towards the end of each week. Irrigated amounts varied greatly (maximum daily value of 42.9 mm) during the irrigation periods. The total measured irrigation was 691.2 mm and 600.4 mm in 2021 and 2022, respectively.

Soil moisture measurements of field S09 are shown in Figure 3 (central panels) for depths of 5, 20, and 50 cm. In general, SM was higher at larger depths, whereas SM dynamics due to events such as precipitation, irrigation, and root water uptake, were more pronounced at shallow depths. Nonetheless, SM at all three measured depths shows a clear response to precipitation and irrigation. Generally, in each year, a period with a high SM is found between November and April, which is followed by a steep decline in SM in the weeks before irrigation starts. This decline is probably related to the high root water uptake from the apple trees and grass in the periods of low precipitation and no irrigation. From mid-May to the end of the irrigation period, SM shows large weekly variations due to the irrigation and precipitation inputs, and due to outputs, such as, in large part, root water uptake from the apple trees and grass. Overall, the average measured SM over the monitored period in field S09 was 0.233, 0.237, and 0.287 cm^3^ cm^−3^ at 5, 20, and 50 cm depth, respectively.

The reference SM and the CRNS-derived SM are shown in the bottom panel of Figure 3. The reference SM is similar to the measured SM at 5 cm depth, due to the weighting procedure that puts higher weights to the shallower depths. Due to instrument installation, update, and maintenance dates, CRNS-derived SM was only available from April to November 2021, and from April to October 2022. The RMSE calculated over the available measurement period was 0.042 cm^3^ cm^−3^. Periods that preceded irrigation showed a relatively low RMSE of 0.025 and 0.018 cm^3^ cm^−3^, except for February and March 2021, where the RMSE was 0.037 cm^3^ cm^−3^. This latter period, however, showed inconsistent neutron counts and was followed by instrument maintenance. Thus, for this period, measurements were disregarded. During the irrigation periods, the RMSE was 0.034 cm^3^ cm^−3^ in 2021 and 0.058 cm^3^ cm^−3^ in 2022, which is considerably higher than in periods of no irrigation. After the 2021 irrigation period, a relatively high RMSE of 0.047 cm^3^ cm^−3^ was found. Such a value is probably due to strong precipitation events that occurred for several days, and may have resulted in a heterogeneous distribution of SM and intercepted water within the CRNS footprint. Unfortunately, this assumption cannot be confirmed, as there was no similar precipitation pattern in 2022. Overall, it appears that the CRNS could provide reliable SM information until the irrigation start. Once irrigation started, the temporal dynamics of SM within the irrigated field were sensed by the CRNS, but a large underestimation of the magnitude of such dynamics is apparent for irrigation events. On the contrary, precipitation events had a larger impact on the neutron count rate dynamics and, when precipitation occurred on multiple consecutive days (e.g., late July 2021), CRNS-derived SM better represented the reference data. Nonetheless, a general underestimation of the magnitude of the SM dynamics was still apparent. Additional details and visual explanations of these effects is proposed in Appendix B.

**Figure 4:**
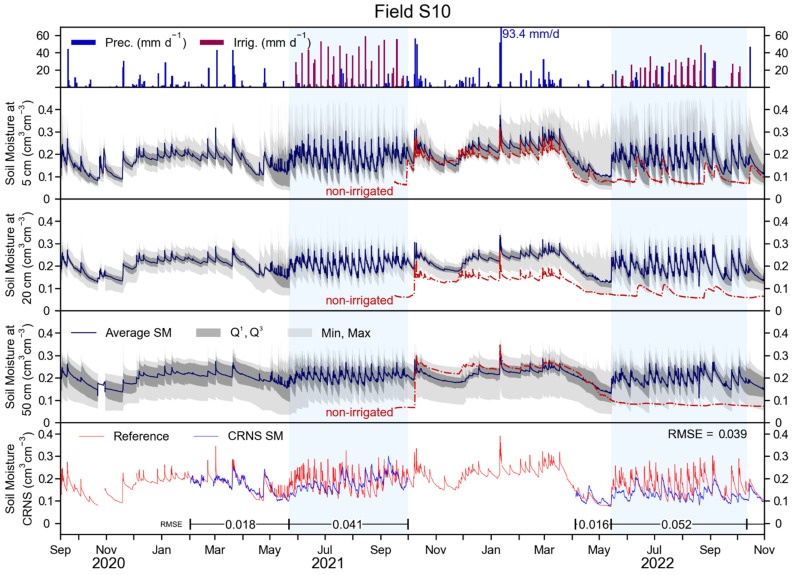
Data recorded in field S10. In the upper panel: precipitation, and irrigation in mm d^−1^. In the three central panels: SM recorded from the SoilNet network at 5, 20, and 50 cm depths. Dark grey areas indicate the first and third quartile, whereas light grey area indicates maximum and minimum values for the SM of these panels. In the bottom panel: reference SM from weighting the SoilNet network and CRNS-derived SM. Regressions of these measurements are provided in Appendix C.

The daily precipitation and irrigation, in mm, for field S10 are shown in Figure 4 (top panel). The irrigation periods started on 15 May 2021 and on 14 May 2022, and ended on 26 September 2021 and on 13 October 2022. Irrigated amounts varied greatly (up to 53.9 mm day^−1^) during the irrigation periods. The total measured irrigation was 897.6 mm and 720.3 mm in 2021 and 2022, respectively, which is 29.9% and 20.0% higher than in field S09.

Soil moisture measurements of field S10 are shown in the central panels of Figure 4 and have similar temporal patterns compared to field S09. However, SM was generally lower in field S10. The average measured SM was 0.181, 0.204, and 0.202 cm^3^ cm^−3^ at 5, 20, and 50 cm depth, respectively, which is 22.3%, 13.9%, and 29.6% lower than in field S09. The red dashed lines show the SM measured by the SoilNet node placed outside the irrigated field (Figure 1). This SM was lower than in the irrigated field, except for the 50 cm depth during winter 2021–2022. The most apparent difference is during the 2022 irrigation period, when the SM was ~0.070 to ~0.080 cm^3^ cm^−3^ in the dry spot in absence of precipitation. Generally, after precipitation, an increase of up to ~0.200 and ~0.140 cm^3^ cm^−3^ was visible at 5 and 20 cm depth, respectively. After the precipitation event, SM outside the irrigated field decreased at a similar rate as in the irrigated field at 5 cm depth, and at a lower rate at 20 cm depth. At 50 cm depth, outside the irrigated field, SM did not clearly respond to precipitation.

The bottom panel of Figure 4 shows the reference SM and the CRNS-derived SM for field S10. Due to instrument installation, update, and maintenance dates, CRNS-derived SM was only available from April to October 2021, and from April to November 2022. The overall RMSE of the CRNS-derived SM was 0.039 cm^3^ cm^−3^ and was lower in periods before irrigation (0.018 and 0.016 cm^3^ cm^−3^ in 2021 and 2022, respectively) compared to irrigation periods (0.041 and 0.052 cm^3^ cm^−3^ in 2021 and 2022, respectively). Similar to field S09, the CRNS could provide reliable SM information until the start of irrigation. During irrigation periods, the temporal dynamics of SM were captured by the CRNS, but the magnitude of the SM dynamics was generally underestimated, as was the case in field S09.

Outside the irrigation period, the CRNSs appear to provide relatively accurate results. During the irrigation period, on the contrary, relatively large deviations, compared to the reference measurements, are found. These deviations can be attributed to the small size of the irrigated fields, compared to the footprint of the CRNSs, which results in the contamination of the CRNS signal by neutrons that originate outside the target field. When the SM in the irrigated field is higher than in the surroundings, a large influx of neutrons that originate outside the field affects the neutron count and results in an underestimation of CRNS-derived SM within the field. This is not the result of an inaccuracy of the CRNS method, but a consequence of the physics behind the CRNS method itself. Nonetheless, it is apparent that the CRNSs could detect the sprinkler irrigation timing, despite the relatively small dimension of the irrigated field, which underlines the relatively high sensitivity of CRNSs with a 25 mm HDPE moderator and gadolinium shield.

### 3.2. Monitoring and Informing Irrigation Practices Using Different Calibrations

**Figure 5:**
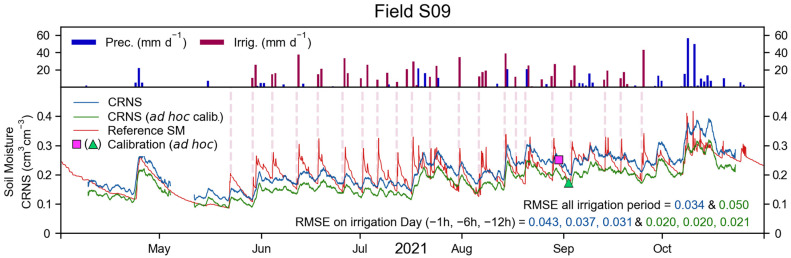
For field S09, during the 2021 irrigation period: precipitation, irrigation, reference SM and CRNS-derived SM obtained with a standard calibration procedure (blue line and pink square) and with an ad hoc calibration procedure (green line and triangle). The RMSE of the entire irrigation period and at one, six, and twelve hours before irrigation is shown in the bottom panel. Irrigation dates selected for the RMSE calculation are marked with red, vertical, dashed lines. Regressions of these measurements are provided in Appendix C.

The precipitation, irrigation, reference SM, and CRNS-derived SM for field S09, during the 2021 irrigation period, are shown in Figure 5. A total of 21 irrigation events took place in field S09. Of these, 20 were recorded by the water meters, whereas one irrigation event was performed before the installation of the monitoring system. These irrigation events generally happened towards the end of each week, but were heterogeneous in time and quantity of water. Nonetheless, irrigation resulted in changes in SM within field S09 that were apparent in the reference SM dynamics (bottom panel of Figure 5). The CRNS-derived SM could partially capture the timing of an irrigation event. However, the magnitude of the SM variation, especially during and immediately after an irrigation event, was underestimated, resulting in a RMSE of 0.034 cm^3^ cm^−3^.

In certain decision-making approaches, SM is not continuously monitored, but only investigated before an irrigation event, to inform on the most suitable irrigation quantity. In such a scenario, CRNS-derived SM could be estimated immediately before irrigation is applied. In the 2021 irrigation period of field S09, the RMSE between reference and CRNS was 0.043, 0.037, and 0.031 cm^3^ cm^−3^ at one, six, and twelve hours before irrigation. If an alternative ad hoc calibration, based on the SoilNet network and performed during a dry period (Figure 5), was used, the general RMSE increased to 0.050 cm^3^ cm^−3^. However, with the ad hoc calibration, the RMSE before irrigation was 0.020, 0.020, and 0.021 cm^3^ cm^−3^ at one, six, and twelve hours before irrigation, which is considerably lower than with a standard calibration approach. With an ad hoc calibration, the use of centred rolling averages with window sizes smaller than 24 h (i.e., 12 and 6 h) results in a higher RMSE. On the contrary, the adoption of a right-aligned rolling average generally results in a lower RMSE, especially in the case of a 12 h window size, for which the RMSE was 0.018, 0.018, and 0.019 cm^3^ cm^−3^ at one, six, and twelve hours before irrigation.

**Figure 6:**
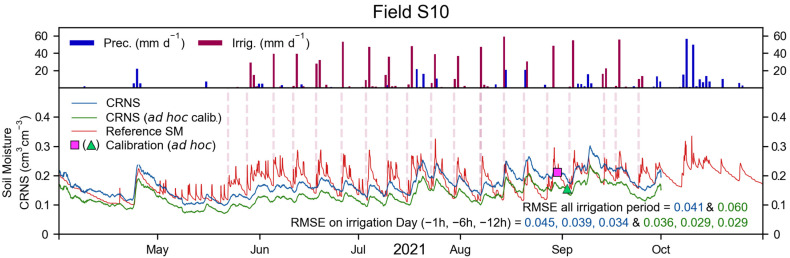
For field S10, during the 2021 irrigation period: precipitation, irrigation, reference SM, and CRNS-derived SM obtained with a standard calibration procedure (blue line and pink square) and with an ad hoc calibration procedure (green line and triangle). The RMSE of the entire irrigation period and at one, six, and twelve hours before irrigation is shown in the bottom panel. Irrigation dates selected for the RMSE calculation are marked with red, vertical, dashed lines. Regressions of these measurements are provided in Appendix C.

The 2021 irrigation period, with precipitation, irrigation, reference SM, and CRNS-derived SM for field S10, is shown in Figure 6. A total of 19 irrigation events took place, of which 18 were recorded by the water meters (first irrigation event occurred before instrument installation). Similar to field S09, irrigation events were generally found towards the end of each week, were heterogeneous in timing and quantity, and resulted in SM dynamics that were apparent in the reference SM (bottom panel of Figure 6). These SM dynamics were only partially captured by the CRNS-derived SM, and their magnitude was underestimated, resulting in a RMSE of 0.041 cm^3^ cm^−3^. If an ad hoc calibration was used, the RMSE of the irrigation period increased to 0.053 cm^3^ cm^−3^. The RMSE at one, six, and twelve hours was 0.045, 0.039, and 0.034 cm^3^ cm^−3^, and 0.036, 0.029, and 0.029 cm^3^ cm^−3^ for the standard and the ad hoc calibration, respectively. With the ad hoc calibration, the use of integration windows of 12 and 6 h resulted in similar or higher RMSE compared to a centred 24 h rolling average. In the case of a right-aligned rolling average, integration windows of 24 and 12 h generally provided a higher RMSE compared to a centred rolling average. On the contrary, a 6 h right-aligned rolling average resulted in a lower RMSE of 0.034, 0.026, and 0.028 cm^3^ cm^−3^, at one, six, and twelve hours before irrigation.

Overall, it is apparent that the CRNS placed in fields S09 and S10 could capture the irrigation timing, but could not adequately represent the SM dynamics that originate from the investigated irrigation practices. If the CRNS was used to investigate SM before irrigation is applied and if an ad hoc calibration was used, the RMSE found at one to twelve hours before an irrigation event was relatively low. This approach may provide sufficiently reliable information on the SM status of the investigated fields, and thus inform decision making in weekly irrigation practices. However, the applicability of this method may be reduced if the irrigation is performed more frequently and with smaller quantities of water, or if a different irrigation method (e.g., drip irrigation) is employed. A relatively lower RMSE was obtained by using a right-aligned rolling average with integration windows shorter than the standard 24 h (i.e., 12 h integration window in field S09 and 6 h in field S10). Even though changes in the RMSE were rather small, these results suggest the possible added value provided by shorter integration windows and by the exclusion of measurements obtained during the irrigation event. Nonetheless, future studies should investigate the added value of employing shorter window sizes compared to the 24 h rolling average (used in this study) and of more complex filtering algorithms in detail [66]. Finally, overall, it is apparent that CRNSs, in this context, are outperformed by the use of a dense sensor network, such as the SoilNet networks employed in this study.

### 3.3. Neutron Transport Simulations

#### 3.3.1. *φ_in_*, *φ_out_*, and $\varphi_{non-albedo}$ from Existing Neutron Transport Simulations

The water retention functions obtained from the Edelman soil samples at 0 to 30 cm provided SM values at field capacity (−100 cm) and at the wilting point (−15,000 cm) of 0.275 and 0.098 cm^3^ cm^−3^ for field S09, and of 0.212 and 0.105 cm^3^ cm^−3^ for field S10 (Table 2). These values were used to interrogate the neutron transport simulations of [35], by using the SM combinations of Table 2 and irrigated field dimensions equal to those of fields S09 and S10. Table 2 shows the resulting $\varphi_{in}$, $\varphi_{out}$, and $\varphi_{non-albedo}$.

#### 3.3.2. $\varphi_{in}$, $\varphi_{out}$, and $\varphi_{non-albedo}$ from Novel Neutron Transport Simulations of the Agia Area

**Figure 7:**
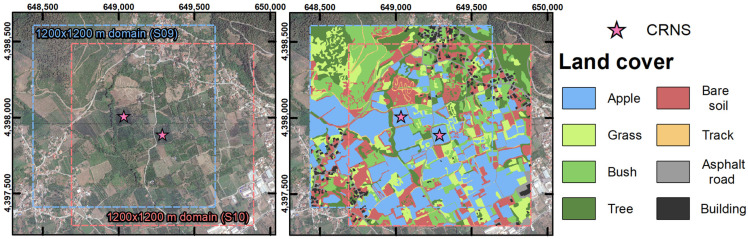
In the left panel: satellite image [75] with the extent of the simulated domains for fields S09 and S10. In the right panel: land cover with 1 m resolution for the investigated areas.

The land use of the study area and the extent of the simulated domains are shown in Figure 7. North of the two CRNSs, natural land use is prevalent (i.e., bare soil, grass, bush, and tree). The southern half of the simulated domains, on the contrary, is dominated by irrigated orchards (apple and cherry). Buildings, asphalt roads, and tracks are found locally and only represent a small portion of the domains. Overall, in the surroundings of the target fields, irrigated fields cover 29.0% and 31.4% of the domain for field S09 and S10, respectively.

The SM that was used in the URANOS neutron transport simulations at depths of 0 to 30, 30 to 60, and 60 to 160 cm is shown in Table 3. These SM values were obtained from SM at field capacity (−100 cm) and at the wilting point (−15,000 cm) of the Edelman samples, as well as from observations of the SoilNet node outside field S10 (Figure 1). Table 3 also shows the $\varphi_{in}$, $\varphi_{out}$, and $\varphi_{non-albedo}$ values obtained from these simulations. Overall, limited differences with the use of existing simplified simulations (Table 2) were found. The average differences are +4.4% for $\varphi_{in}$, −3.9% for $\varphi_{out}$, and −0.5% for $\varphi_{non-albedo}$.

These results suggest that there is a difference between φ values obtained by using (a) existing simplified neutron transport simulations, that do not consider vegetation and have homogeneous vertical and horizontal SM distributions and (b) a more accurate representation of the simulated area. Although differences between φ values obtained with these two methods were rather small in the studied area, they could be more pronounced in different environments. For example, when the target irrigated field has an elongated or irregular shape or when SM, inside or outside the irrigated field, is extremely heterogeneous in space [35].

### 3.4. CRNS Correction with SM Outside the Irrigated Field

**Figure 8:**
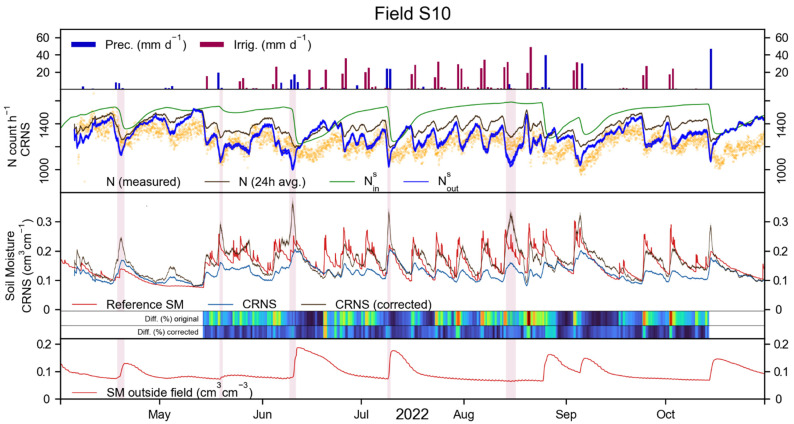
In the first and second panel, for field S10 during the 2022 irrigation period: precipitation, irrigation, uncorrected and corrected neutron count, and synthetic neutron counts $N_{in}^{s}$. and $N_{out}^{s}$. In the third panel: reference SM, standard CRNS-derived SM (blue line), and corrected CRNS-derived SM (black line). The coloured horizontal bars show the difference between CRNS and the reference SM (in % of the reference value), with blue for low difference and red for high difference. In the fourth panel: SM recorded by the sensor outside field S10.

The top panel of Figure 8 shows precipitation and irrigation in field S10 during the 2022 irrigation period. The second panel of this figure shows the measured neutron count rate (orange dots) and the neutron count rate corrected for $C_{p}$, $C_{h}$, $C_{v}$, $C_{i}$, and 24 h rolling average (brown line) of the CRNS in field S10. The third panel shows the reference SM (red line) and the CRNS-derived SM (blue line), for which the RMSE during the 2022 irrigation period was 0.052 cm^3^ cm^−3^. The CRNS representation of the reference data was similar to that of the 2021 irrigation periods (Figure 5 and Figure 6), with the CRNS able to capture the irrigation timing, but showing considerable underestimation of the magnitude of the SM dynamics. This is caused, primarily, by the small area of the irrigated field, which results in the detection of a large number of neutrons that originate and carry information from the surroundings of the target field. The second panel of Figure 8 also shows the synthetic neutron count $N_{out}^{s}$ (green line), that is obtained from the SM of the SoilNet outside the irrigated area (bottom panel of Figure 8). Blue lines show the synthetic $N_{in}^{s}$ estimated with the proposed CRNS correction approach and with the subdivision of N (i.e., $\varphi_{in}$, $\varphi_{out}$, and $\varphi_{non-albedo}$) from the neutron transport simulations of the Agia area. The two $N_{in}^{s}$ are used to obtain two corrected SM values (minimum and maximum) that are shown with a grey area in the third panel of Figure 8. These two values are rather similar and, in general, their difference does not exceed 0.020 cm^3^ cm^−3^. A black line shows the average between the two corrected CRNS-derived SM values.

The corrected CRNS better captured the timing and magnitude of the reference SM dynamics, which is also reflected by a 0.036 cm^3^ cm^−3^ RMSE (lower than the uncorrected case). Nonetheless, the corrected SM locally underestimated the reference data like the uncorrected SM (e.g., in mid-June) and sometimes overestimated the reference data (red areas in Figure 8). In the case of overestimation, it can be observed that the SM measured by the SoilNet node outside the irrigated field either did not react to precipitation or showed a delay between precipitation and SM increase. Such an effect could be due to the 5 cm depth at which the shallower sensor was positioned. This affects the CRNS correction procedure by artificially increasing $N_{out}^{s}$, and thus decreasing $N_{in}^{s}$, which finally results in an overestimation of SM. If these overestimation periods (red areas in Figure 8) are not considered, the RMSE of the uncorrected CRNS-derived SM remained 0.052 cm^3^ cm^−3^ and decreased to 0.031 cm^3^ cm^−3^ for the corrected, CRNS-derived SM. The use of existing synthetic neutron transport simulations to retrieve the subdivision of N (i.e., $\varphi_{in}$, $\varphi_{out}$, and $\varphi_{non-albedo}$) in field S10 resulted in a relatively higher RMSE of 0.043 cm^3^ cm^−3^, which was reduced to 0.033 cm^3^ cm^−3^ if the overestimation periods were not considered. This last RMSE obtained with existing neutron transport simulations was only 0.002 cm^3^ cm^−3^ (6.5%) higher than that obtained with new simulations of the study area. Furthermore, a visual inspection of the results shows that the two corrected SM similarly represent the dynamics of the reference data (see Appendix D).

The correction procedure was also tested on field S09, even though this field was not equipped with a nearby SM sensor. Thus, the SM sensor outside field S10 was used for field S09. Different results compared to those of field S10 were obtained and a relatively large overestimation was found. This overestimation was attributed to the large distance between the CRNS in field S09 and the supporting SoilNet sensor (~250 m, see Figure 1), as well as the different SM conditions within and in the surroundings of field S09, compared to field S10. In fact, as shown in Figure 3 and Figure 4, SM in field S10 was up to 29.6% lower than in field S09 and irrigation in field S10 was up to 29.9% higher than in field S09. Thus, the SM measured outside field S10 was increased by 0.050 cm^3^ cm^−3^, which is the difference between the average SM at 5 cm depth between field S09 and field S10. Consequently, the corrected CRNS-derived SM better matched the reference SM (RMSE of 0.049 cm^3^ cm^−3^) compared to the uncorrected CRNS-derived SM (RMSE of 0.058 cm^3^ cm^−3^). More important than the RMSE values, the corrected CRNS-derived SM offered a relatively improved representation of the reference SM in terms of timing and magnitude of SM dynamics. These findings are discussed in more detail in Appendix E. Overall, results suggest that a supporting SM sensor should be placed nearby the target irrigated field, as it can be challenging to correct multiple CRNSs of a heterogeneous agricultural area with a single supporting SM sensor. Although it can be argued that an improved correction for field S09 could be obtained if a SM sensor was installed directly outside this field, further research is needed to corroborate these results and hypothesis.

Overall, the corrected CRNS-derived SM in field S10 provided superior performance compared to the uncorrected CRNS-derived SM, and was relatively similar to the results obtained with a dense sensor network. Some limited overestimation periods, generally caused by a delay in the recording of SM changes due to precipitation, do not seem to undermine the general positive results of the proposed methodology. This correction approach could, thus, prove valuable in irrigation monitoring with CRNSs when a dense sensor network is either too costly, or too complex to install and maintain. Although the use of neutron transport simulations that are representative of the study area resulted in the lowest RMSE, the use of existing simplified simulations still provided valuable results. In the specific case of S10, the additional effort required to perform these simulations could be saved without losing a significant amount of precision in the final SM.

### 3.5. Limitations of the CRNS Correction Approach and Outlook

Despite the positive results of this study, different shapes of the irrigated field could prove more challenging to monitor with the proposed correction of CRNS measurements. For example, the effectiveness of the correction method may be reduced in fields with elongated or irregular shapes, as this can influence the origin of the detected neutrons [35]. In this case, the use of neutron transport simulations of the target area could provide additional benefits compared to existing simulations. However, when the irrigated field is particularly small (e.g., 0.5 ha or less), it should be further investigated if CRNS is outperformed by the use of point-scale sensors or of instruments with a smaller footprint (e.g., gamma-ray spectroscopy [87]).

It is also possible that the proposed correction of CRNS-derived SM will not be suitable for all irrigation methods and scenarios. For example, irrigation techniques that result in small variations of the SM in the irrigated field might pose additional challenges. This is the case in drip irrigation, where the SM changes are limited to the portions of the irrigated field [65]. Thus, further research is necessary to validate the findings of this study and to verify to which extent they are applicable. Future studies could focus on different dimensions of the irrigated area and test smaller fields compared to those investigated in this work.

The correction proposed In this study is rather complex in its current form. Using a dataset of available neutron transport simulations represented a considerable simplification and provided results that were comparable to those obtained with new simulations of the study area. Nonetheless, future studies should verify if specific simulations could be necessary in areas with a higher degree of environmental heterogeneities or with rougher and more discontinuous topography. Although the application of the current correction procedure is challenging for the end user, it could be framed into a commercial decision support system, as is the case for several other SM monitoring devices used in irrigation scheduling.

In this study, the proposed correction of CRNS-derived SM was effective when the supporting SM sensor was located nearby the target field (i.e., field S10). On the contrary, poorer performance was found in a relative distant field (i.e., field S09), which may be due to differences in soil properties and SM patterns compared to the supporting SM sensor location. It can be thus argued that improved results would be obtained if the soil properties and SM patterns of field S09 and S10 were relatively similar. Nonetheless, the possibility to use a single SM sensor (or a set of spatially distributed sensors) to correct multiple CRNSs of an irrigated area should be further investigated.

The influence of strong vertical heterogeneity in SM, especially in the top cm of soil, represent an additional limitation and should be further addressed in future studies. Additionally, the use of the universal transport solution [28], in place of the standard approach of [26], should be tested in irrigation scenarios. Moreover, the influence of air humidity, vegetation cover, and different moderators and shielding should be further studied, as these can result in the detection of neutrons with different energy and origin.

Finally, although CRNSs could replace a sensor network, and thus lower costs in a variety of scenarios, price is known to be one of the factors limiting a widespread adoption of SM monitoring tools by farmers. However, in the upcoming years, lower prices due to instrument improvements, larger standardized production, employment in high-value agricultural settings, and subsidization of farms that use water-saving technologies could strongly enhance the adoption of SM monitoring tools in general.

## 4. Conclusions

This study investigates CRNS monitoring of SM in two irrigated orchards (fields S09, 1.25 ha, and field S10, 1.17 ha) in Greece, by comparing CRNS-derived SM to a dense sensor network. The comparison was performed during the irrigation periods of 2021 and 2022. Results for the 2021 irrigation period showed that a CRNS with a 25 mm HDPE moderator and a gadolinium-oxide-based thermal shield could identify the timing of weekly irrigation events. However, it was not possible to capture the magnitude of irrigation-related SM dynamics. This was attributed to the relatively small size of the irrigated fields, which results in the detection of neutrons that originate and carry information from the field’s surroundings. When measurements alone, obtained at one, six, and twelve hours before irrigation, were considered, the CRNSs could provide more reliable information. This was especially the case when an ad hoc calibration, based on the SoilNet network during a dry period, was used, which resulted in an RMSE of 0.020, 0.020, and 0.021 in field S09, and 0.035, 0.028, and 0.029 in field S10. The use of a right-aligned rolling average with shorter integration window than the standard 24 h sometimes resulted in a lower RMSE. A right-aligner 12 h rolling average resulted in an RMSE between 0.018 and 0.019 in field S09, and a 6 h one resulted in an RMSE between 0.036 and 0.029 in field S10. Although these results show that CRNSs could be used to monitor SM in the hours before an irrigation event, different irrigation strategies could pose a greater challenge, for example, when irrigation is applied more frequently than in the investigated fields.

During the 2022 irrigation period, an additional SM sensor was installed directly outside field S10. A correction of the neutron count and of the CRNS-derived SM based on this sensor was applied. For this, neutron transport simulations of the study area were produced to obtain subdivisions between the detected neutrons that originated in the irrigated field, outside the irrigated field, and that had no soil contact. These subdivisions were used to correct the CRNS signal. The proposed correction reduced the RMSE of the CRNS-derived SM from 0.052 to 0.036, and then to 0.031 when periods of clear overestimation were discarded. More importantly, the use of the proposed correction procedure resulted in CRNS-derived SM, that clearly represented both the timing and the magnitude of the reference SM dynamics for the irrigated field. The use of simplified simulations to obtain the subdivisions of detected neutrons resulted in a somewhat higher RMSE. In the case of field S09, the proposed correction using the SM sensor outside field S10 resulted in relatively large overestimation of SM. This overestimation was substantially reduced when the difference in average SM between field S09 and field S10 was considered. Nonetheless, results suggest that, to obtain optimal results, the non-irrigated SM sensor may have to be located in a soil with similar physical properties as the target field immediate surroundings.

Overall, this study shows that the novel correction procedure for CRNS-derived SM is clearly beneficial for the monitoring of SM in small, irrigated fields. Future research should further validate the results of this work and verify the applicability of the proposed correction to fields with different dimensions, shapes, and surroundings, as well as to different irrigation practices. In addition, the possibility of using a limited number of SM sensors to correct multiple CRNSs in fields of a large agricultural area could be tested. The results of this study are a further step towards the use of CRNSs as a decision support system in irrigation management, and thus towards a more efficient use of water resources in agriculture.

## Figures and Tables

**Table 1:** SM combinations of the four neutron transport simulations used to perform the proposed CRNS correction method.

Simulated Scenario SM (in–out)	$\theta_{in}$	$\theta_{out}$
dry–dry	$\theta_{in\left(min\right)}$	$\theta_{out\left(min\right)}$
dry–wet	$\theta_{in\left(min\right)}$	$\theta_{out\left(max\right)}$
wet–dry	$\theta_{in\left(max\right)}$	$\theta_{out\left(min\right)}$
wet–wet	$\theta_{in\left(max\right)}$	$\theta_{out\left(max\right)}$

**Table 2:** Combinations of SM values $\theta_{in}$ and $\theta_{out}$ used to interrogate the existing neutron transport simulations, and the resulting subdivision of N (in%).

Field	Scenario (in–out)	$\theta_{in}$ (cm^3^ cm^−3^)	$\theta_{out}$ (cm^3^ cm^−3^)	$\varphi_{in}$ (%)	$\varphi_{out}$ (%)	$\varphi_{non-albedo}$ (%)
S09	dry–dry	0.098	0.070	46.5	38.9	14.6
	dry–wet	0.098	0.200	54.1	29.1	16.8
	wet–dry	0.275	0.070	39.7	42.8	17.6
	wet–wet	0.275	0.200	47.0	32.1	20.9
S10	dry–dry	0.105	0.070	45.5	39.8	14.7
	dry–wet	0.105	0.200	53.0	29.8	17.2
	wet–dry	0.212	0.070	40.9	42.5	16.6
	wet–wet	0.212	0.200	47.8	32.4	19.8

**Table 3:** SM values used to produce new URANOS neutron transport simulations using combinations of $\theta_{in}$ and $\theta_{out}$, and the resulting subdivision of N (in%).

Field	Scenario (in-out)	$\theta_{in}$ (cm^3^ cm^−3^) for 30–60–160 cm Depth	$\theta_{out}$ (cm^3^ cm^−3^) for 30–60–160 cm Depth	$\varphi_{in}$ (%)	$\varphi_{out}$ (%)	$\varphi_{non-albedo}$ (%)
S09	dry-dry	0.098–0.093–0.093	0.070–0.080–0.080 ^1^	51.1	34.3	14.6
	dry-wet	0.098–0.093–0.093	0.200–0.100–0.100 ^1^	57.3	26.7	16.0
	wet-dry	0.275–0.245–0.221	0.070–0.080–0.080 ^1^	45.6	36.6	17.8
	wet-wet	0.275–0.245–0.221	0.200–0.100–0.100 ^1^	50.4	30.0	19.6
S10	dry-dry	0.105–0.114–0.114	0.070–0.080–0.080 ^1^	50.6	34.5	14.9
	dry-wet	0.105–0.114–0.114	0.200–0.100–0.100 ^1^	56.6	26.9	16.5
	wet-dry	0.212–0.214–0.191	0.070–0.080–0.080 ^1^	46.7	36.8	16.5
	wet-wet	0.212–0.214–0.191	0.200–0.100–0.100 ^1^	51.5	30.2	18.3

^1^ The $\theta_{out}$ in the surrounding irrigated fields was set equally to the average of fields S09 and S10. At 0 to 30, 30 to 60, and 60 to 160 cm depths, $\theta_{out}$ was 0.101, 0.103, and 0.104 cm^3^ cm^−3^ in dry conditions, and 0.243, 0.229, and 0.222 cm^3^ cm^−3^ in wet conditions.

**Figure A1:**
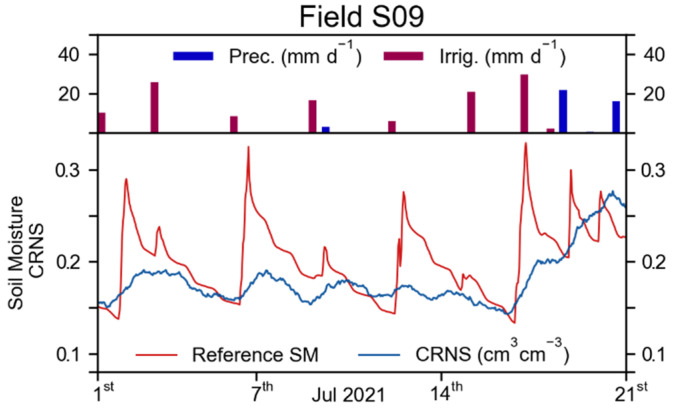
Text for field S09 between 1 and 21 July 2021: precipitation, irrigation, reference SM, and CRNS-derived SM.

**Figure A2:**
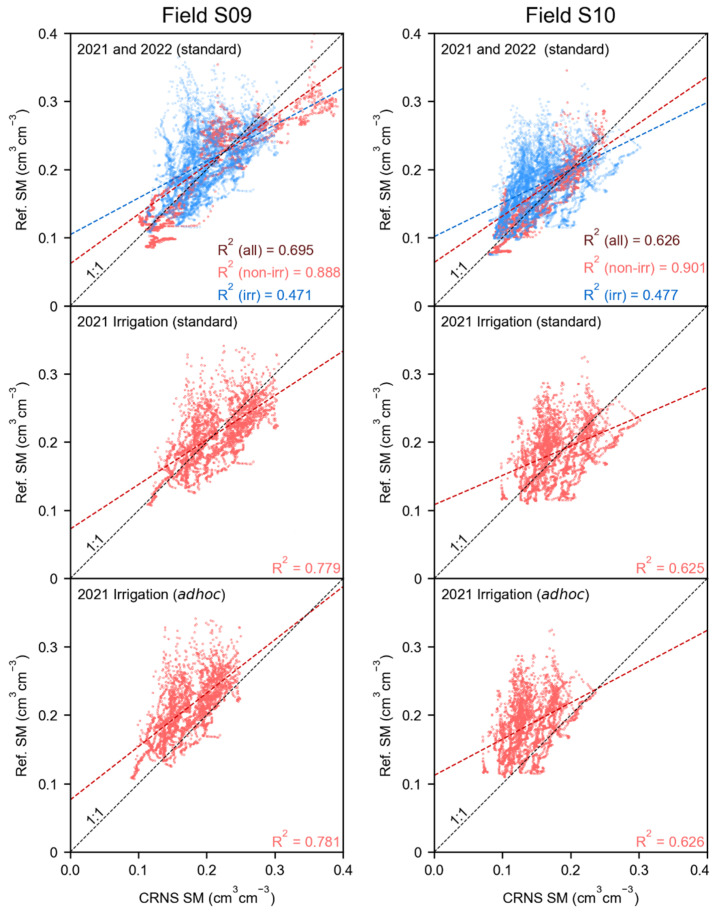
Regressions between the reference SM from SoilNet, and the CRNS-derived SM for fields S09 and S10. The upper panels show the entire measurement period with R^2^ for all available measurements (all), non-irrigated (non-irr), and irrigated (irr) periods. Central panels show the use of a standard calibration for the 2021 irrigation, whereas bottom panels show the use of an ad hoc calibration for the same period.

**Figure A3:**
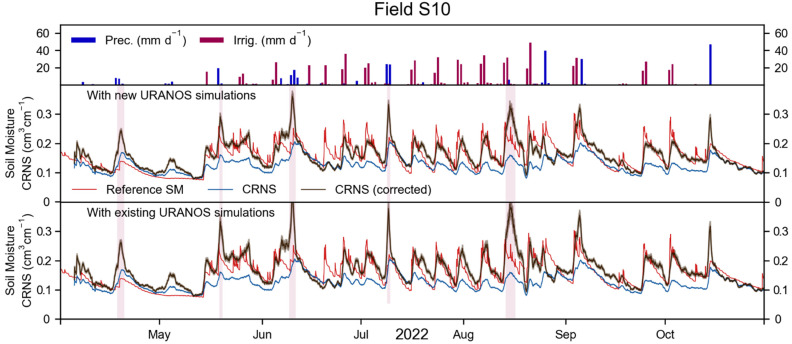
In the first panel precipitation and irrigation for field S10 during the 2022 irrigation period are provided. In the second panel, reference SM, standard CRNS-derived SM (blue line), and corrected CRNS-derived SM (black line) that used new URANOS simulations are shown. In the third panel, the use of existing URANOS simulations for the correction of CRNS-derived SM is shown.

**Figure A4:**
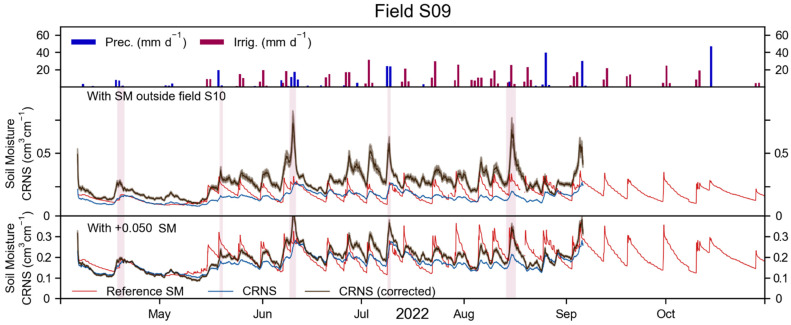
In the first panel, precipitation for field S10 and irrigation for field S09 during the 2022 irrigation period are shown. In the second panel, reference SM, standard CRNS-derived SM (blue line), and corrected CRNS-derived SM (black line), that used SM values from a sensor outside S10, are shown. In the third panel, results of the CRNS correction, if SM of the sensor outside field S10 is increased by 0.050 cm^3^ cm^−3^, are shown.

## Data Availability

Data are available upon contacting the authors.

## Supplementary Materials

The following supporting information can be downloaded at: https://www.mdpi.com/article/10.3390/s23052378/s1, Spreadsheet1: CRNS_Correction_DIY.

## Appendix A

The hydraulic parameters were estimated using the PTFs provided by Rawls and Brakensiek [73], which use the soil bulk density and the fractions of sand, silt, and clay. Using the method of Brakensiek and Ralws [72], the fraction of gravel was used to estimate the bulk density of the soil $BD_{t}$ using:

(A1) $$BD_{t}=BD_{<2}+G_{v}\left(BD_{>2}-BD_{<2}\right),$$

where $BD_{<2}$ is the bulk density of the fine soil, which was assumed to be equal to 1.3 g cm^−3^, $BD_{>2}$ is the bulk density of the gravel fraction, which was assumed to be equal to 2.65 g cm^−3^ (solid quartz), and $G_{v}$ is the volume of the gravel. $G_{v}$ was obtained from the method of Flint and Childs [88], using:

(A2) $$G_{v}=G_{w}/\left(2-G_{w}\right),$$

where $G_{w}$ is the weight fraction of the gravel.

## Appendix B

Figure A1 shows precipitation, irrigation, reference SM, and CRNS-derived SM in field S09 from the 1 July to the 21 July 2021. The reference SM reacted quickly to irrigation events and SM increased by more than ~0.015 cm^3^ cm^−3^, before rapidly decreasing until the subsequent irrigation event. The CRNS-derived SM increased due to an irrigation event, but in a less abrupt way compared to the reference SM, which was probably due to the use of a 24 h rolling average. The CRNS-derived SM, despite showing a timing of the SM increase that is similar to that of the reference SM, only showed ~0.030–0.040 cm^3^ cm^−3^ variations from 1 July to 17 July. This underestimation was due to the small area (1.25 ha) over which irrigation was applied, and the influence of the field surroundings, where SM was generally lower due to the absence of irrigation and precipitations. When precipitation occurred on 18 July, SM was increased over the entire sensing volume of the CRNS, which resulted in a large increase in the CRNS-derived SM.

## Appendix C

For fields S09 and S10, Figure A2 shows the results of regressions performed using reference SM values and CRNS-based SM. The upper panels of Figure A2 show the entire available measurement period. The coefficient of determination (R^2^) is higher in the non-irrigated periods (0.888 and 0.901 in fields S09 and S10, respectively) compared to the irrigated periods (0.471 and 0.477). The central panels of Figure A2 show the use of a standard calibration for the 2021 irrigation period, whereas the bottom panels show the use of an ad hoc calibration for the same period. The use different calibration strategies results in different regression lines, but does not result in large variations of the R^2^ (e.g., for field S09, 0.779 and 0.781, with a standard and an hoc calibration, respectively).

## Appendix D

Figure A3 shows the difference between the use of the new neutron transport simulations of field S10 (second panel) and the use of existing, simplified simulation results (third panel). The RMSE of the former was 0.036 cm^3^ cm^−3^, whereas the RMSE with the existing simulations was 0.042 cm^3^ cm^−3^. If periods of overestimation (red areas) were removed, the RMSE decreased to 0.031 and to 0.033 cm^3^ cm^−3^. Despite such a difference, both corrected CRNS-derived SMs provide a good representation of the reference SM (timing and magnitude of the dynamics), and are superior compared to the uncorrected CNRS-derived SM.

## Appendix E

In field S09, the use of a correction, based on the SM measured outside field S10, resulted in a large overestimation of the SM (second panel in Figure A4). The reason for such an overestimation could be the distance between the CRNS in field S09 and the SM sensor used for the correction procedure (~250 m). This assumption is supported by the different soils, as well as by the different SM dynamics found in fields S09 and S10 (Figure 3 and Figure 4). In particular, the average SM in field S09 was higher than in field S10 by 0.050, 0.033, and 0.085 cm^3^ cm^−3^ at 5, 20, and 50 cm, respectively.

The RMSE of the uncorrected CRNS-derived SM during the 2022 irrigation period was 0.058 cm^3^ cm^−3^. The RMSE of the corrected CRNS was 0.125 cm^3^ cm^−3^ (second panel of Figure A4), but decreased to 0.051 cm^3^ cm^−3^ if the SM outside field S10 (used for the correction) was increased by 0.050 cm^3^ cm^−3^ (third panel of Figure A4). Such an increase in SM is equal to the difference between the average SM at 5 cm in fields S09 and S10. When existing, simplified neutron transport simulations were used instead of simulations of the study area, the RMSE was 0.049 cm^3^ cm^−3^. More important than the RMSE values, the corrected CRNS-derived SM of the third panel of Figure A4 offers a superior representation of reference SM dynamics than the uncorrected CRNS-derived SM, both in terms of timing and magnitude of SM dynamics.

These findings suggest that improved results for field S09 could be obtained if a SM sensor was installed directly outside this field. However, further research is needed to corroborate these results and hypothesis.
